# Continuous Glucose Monitoring in Resource-Constrained Settings for Hypoglycaemia Detection: Looking at the Problem from the Other Side of the Coin

**DOI:** 10.3390/bios8020043

**Published:** 2018-04-25

**Authors:** Rubao Bila, Rosauro Varo, Lola Madrid, Antonio Sitoe, Quique Bassat

**Affiliations:** 1 Centro de Investigação em Saúde de Manhiça (CISM), CP1929 Maputo, Mozambique; rubao.bila@manhica.net (R.B.); Rosauro.Varo@manhica.net (R.V.); lola.madrid@isglobal.org (L.M.); antonio.sitoe@manhica.net (A.S.); 2 ISGlobal, Hospital Clínic—Universitat de Barcelona, 08036 Barcelona, Spain; 3 Institució Catalana de Recerca i Estudis Avançats (ICREA), Pg. Lluís Companys 23, 08010 Barcelona, Spain; 4 Pediatric Infectious Diseases Unit, Pediatrics Department, Hospital Sant Joan de Déu (University of Barcelona), 08950 Barcelona, Spain

**Keywords:** continuous glucose monitoring, hypoglycaemia, low-income countries

## Abstract

The appearance, over a decade ago, of continuous glucose monitoring (CGM) devices has triggered a patient-centred revolution in the control and management of diabetes mellitus and other metabolic conditions, improving the patient’s glycaemic control and quality of life. Such devices, the use of which remains typically restricted to high-income countries on account of their elevated costs, at present show very limited implantation in resource-constrained settings, where many other urgent health priorities beyond diabetes prevention and management still need to be resolved. In this commentary, we argue that such devices could have an additional utility in low-income settings, whereby they could be selectively used among severely ill children admitted to hospital for closer monitoring of paediatric hypoglycaemia, a life-threatening condition often complicating severe cases of malaria, malnutrition, and other common paediatric conditions.

## 1. Introduction

The advent of technological developments has allowed, in recent years, a significant improvement in the management of diabetes mellitus (DM) globally, including the development of a plethora of point-of-care (PoC) and point-of-need technologies. These have, on account of their simplicity, immediateness of results, precision, and reliability, simplified and democratized the screening, management, and follow-up of this condition. Examples of these devices include improved blood glucose meters, glycated haemoglobin A1c PoC tests, blood betahydroxybutyrate analysers, full lipid panel biochemical PoC, and semi-quantitative urine dipstick or fully automated urinalysis PoC methods [1]. Continuous glucose monitoring (CGM) devices, and the possibility of linking them to new “wearable” insulin delivery systems or pumps, have revolutionized the management of DM, allowing for more patient-centred and intensive monitoring of glycaemia values and trends, improving the knowledge-based capacity to adjust therapeutic dosing.

The CGM approach presents some additional interesting advantages. Current recommendations for assessing glycaemic control involve the periodic monitoring of glycated haemoglobin (HbA1c), a method reflecting average plasma glucose over the preceding 8–12 weeks [2]. This is a relatively simple laboratory-based method, not requiring any special preparation such as fasting. This method can be performed at any time of the day. However, HbA1c levels only reflect average glycaemia concentrations, and cannot characterize day-to-day glycaemic fluctuations or particular excursions (hypoglycaemia, postprandial hyperglycaemia) that may be at the root of acute events or potential diabetes-associated complications. CGM devices could address, at least partially, some of the limitations related to self-monitoring of blood glucose and HbA1c testing, outperforming HbA1c as the sole marker of glycaemic control and thus also facilitating a real-time evaluation of time spent in the target glucose range [3]. CGM devices have also partially removed the nuisance of continuous blood-pricking, providing an uninterrupted and close evaluation of interstitial glucose levels, an acceptable proxy measurement of real glycaemia [4]. Although their use requires the initial insertion of the sensor under the skin (which is a relatively simple and automatized procedure using the commercial products available), the devices can provide continuous and real-time readings for up to a week at a time [3,5].

For all of the aforementioned reasons, the uptake of CGM devices in high-income countries (HICs) is swiftly increasing, with good acceptability and favourable evaluation from end-users [6,7], and an overall impression that they empower patients and contribute to a better control of the disease. The high costs associated with using these devices, particularly if having to privately purchase them, remain a major barrier to wider use.

## 2. Glycaemia-Associated Problems in Low-Income Countries

### 2.1. The Problem of Diabetes Mellitus in Resource-Constrained Settings

The escalating global diabetes epidemic that the 21st century is witnessing is one of the major tribulations of the global health community. The burden and impact of diabetes mellitus (DM) in resource-constrained settings is far from being adequately characterized, but recent estimates suggest that DM has increased faster there than in more industrialized settings, both in terms of its general prevalence and also in the overall number of adults affected [8], possibly as a result of the inadequacies and shortfalls of current prevention and control programs. Indeed, the number of adults living with DM, which has quadrupled in the last quarter of a century globally, appears to be now rapidly increasing not only in rich settings, where this disease has traditionally found a niche, but also among those populations in low and middle-income countries (LMICs) progressing through a demographic transition, and is directly linked with other risk factors associated with poor health outcomes. One of the major caveats of the new technological revolution in regards to DM care is, despite its enormous potential, the slow penetration of new PoC devices and diagnostic and therapeutic aids in LMICs, possibly in relation to their current prohibitive costs. In rural areas of low-income countries, and particularly in Sub-Saharan Africa, where qualified human resources in the health system are scarce and laboratory infrastructures and diagnostic tool availability are very limited, challenges for DM prevention and control become dramatically blatant, as standard management of diabetes is poor or non-existent. Thus, in these settings, and recognizing that many other pressing health priorities and access inequities still need to be resolved, urgent action is required to increase the availability of DM therapeutics and diagnostic aids, and also to expose the huge uncovered burden of metabolic disease, allowing more feasible and targeted policy recommendations which can be implemented to impact the burden and prognosis of DM.

### 2.2. The Burden, Impact, and Risk-Factors for Hypoglycaemia in Low-Income Settings

In LMICs, hypoglycaemia appears as a significant albeit poorly recognized public health problem. The definition of hypoglycaemia implies the detection in an individual of an abnormally low level of blood sugar. Hypoglycaemia thresholds in children, as defined by the World Health Organization, depend on nutritional status, and usually entail a glycaemia level <2.5 mmol/L (45 mg/dL) in an adequately-nourished child, or <3 mmol/L (54 mg/dL) in a severely malnourished child [9]. Current screening of hypoglycaemia at the bedside of the patient is generally conducted by means of simple and relatively inexpensive bedside glucometers, which provide an immediate and actionable measurement at any given point of interest. Their generalized use, including in low-income settings, has allowed for the characterization of hypoglycaemia as a major and increasingly acknowledged risk factor for morbidity and mortality, particularly in developing settings. As a paradigmatic example, the prevalence of hypoglycaemia among paediatric admissions in Sub-Saharan Africa has been estimated to range between 1.8% and 7.3% [10,11]. The consequences of severe or prolonged hypoglycaemia are dreaded, as this condition can result in significant morbidity, for example recurrent seizures, neurological deficits, or even mental retardation [12,13]. Indeed, the appearance of hypoglycaemia in the course of any severe condition is often considered a proxy of the severity of illness, and a harbinger of an adverse outcome, although it is still not well understood how proactive preventive strategies for hypoglycaemia could impact the risk of death. Hypoglycaemia may affect both children and adults (including pregnant women) [14,15,16], with newborns and malnourished children being the most vulnerable groups [17,18,19]. A study describing the prevalence and incidence of hypoglycaemia for all-cause admissions among a large cohort of nearly 50,000 children admitted to a rural Mozambican hospital during a 13-year long period reported an overall high prevalence of hypoglycaemia (3.2%), rising to 8.8% when considering the neonatal period alone. Particularly worrying was the excessive and unacceptable risk of death associated to hypoglycaemia, considering this complication is easily and readily treatable, with nearly one of every five patients affected dying. Importantly, such results are likely an underestimation of the true burden of impact, as glycaemia in this cohort was only detected through a single screening upon admission, and not throughout the rest of the hospitalization [20,21]. Unfortunately, this a common strategy in resource-constrained settings where one aims to make best use of the few tests available.

In resource-constrained settings, hypoglycaemia typically appears in the context of specific conditions such as malaria, diarrhoea, or malnutrition, or as a result of other life-threatening conditions (including meningitis and sepsis) or situations (i.e., the neonatal period) often associated with glycaemia homeostasis disturbances [11,22,23,24,25,26,27].

In the neonatal period, hypoglycaemia is a significant cause of disease and death, and lack of detection or improper management can lead to severe neurological sequelae or lethal outcomes [17,18]. As newborns rely on their mothers for feeding, any common neonatal health problem, including prematurity, birth asphyxia, or infection, may hinder the newborn’s capacity to feed, thus impairing proper nutrition. Newborns are therefore highly vulnerable to blood glucose homeostasis impairments, although the true burden of hypoglycaemia among newborns from developing settings remains enigmatic as routine control of glycaemia is seldom assessed.

Malaria is a well-known cause of hypoglycaemia. The underlying mechanisms for it are multifactorial, including, among others, an excessive consumption of glucose by the *Plasmodium* parasite, the hyperinsulinaemic adverse effect resulting from the use of certain antimalarials (for instance quinine, typically in pregnancy), and the insufficient or inadequate supplementation/oral intake in cases of severe disease, particularly in cerebral malaria cases [24,27,28,29]. Indeed, hypoglycaemia is a defining feature of severe malaria [9], often accompanying other complications such as acidaemia/hyperlactataemia [30]. In severe malaria patients, it is also a treatable cause of coma and convulsions [9]. The prevalence of malaria-associated hypoglycaemia seems to vary in different parts of the world, but can occur in up to a quarter of hospitalized malaria patients [31]. The prognostic implications of hypoglycaemia as part of severe malaria can be easily understood when comparing the mortality risk in patients with (associated case fatality rates (CFR) of 24–61.5%) or without (CFR 8–13.4%) this complication [32,33,34].

Another condition typically associated to an increased risk of hypoglycaemia is severe malnutrition. It has been estimated that malnutrition significantly contributes to at least one third of all global child deaths and the presence of hypoglycaemia is potentially a major contributing factor to such a poor prognosis [19]. In these patients, several factors may compromise glucose homeostasis, such as for instance a shortage in the exogenous nutritional intake, the reduced absorption of sugars resulting from intestinal villous atrophy, or a generalized augment in oxidative stress. The rigorous application of the WHO management of malnutrition guidelines significantly improves the prognosis in these patients, likely in relation to the fact that these guidelines take into special consideration the prevention and early treatment of sepsis and hypoglycaemia [35].

### 2.3. Continuous Glucose Monitoring for Hypoglycaemia Detection in Severely Ill Hospitalized Children in Low-Income Settings

Very little research has been conducted in LMICs specifically exploring the utilization of continuous glucose monitoring for the screening and monitoring of hypoglycaemia. This is not a surprise as bedside glucometers remain much more cost-effective to screen for this complication, and current costs do not support the routine use of CGM in these settings. However, these devices have been used for research purposes, with the aim of better characterizing the burden of recurrent hypoglycaemia among severely ill children during hospitalization. A study among 74 malaria paediatric patients in Southern Mozambique concluded that CGM was a reliable and clinically accurate tool to evaluate blood glucose levels in paediatric malaria patients, and that hypoglycaemia episodes beyond admission in such patients appeared to occur significantly more commonly than previously considered. In this study, the insertion of a CGM device during the initial and most critical days of admission allowed for enhanced detection of low glycaemia episodes, in addition to a more robust and continuous characterization of the dynamics and evolution of blood glucose. The CGM instrument could thus be selectively utilized to better monitor those severely ill patients at higher risk of developing life-threatening hypoglycaemia episodes, allowing for more precise targeting of glucose supplementation and closer observation to improve prognosis during the hospital stay. Studies among other vulnerable populations in low-income settings are now underway, with the aim of assessing the utility of CGM systems among sick neonates and severely malnourished children, two high-risk populations for developing hypoglycaemic episodes.

It will be important to determine whether the particularities of these two populations in terms of fat tissue body composition will allow for the correct functioning and accuracy of the sensors, and therefore reliable monitoring of the outcome. Additionally, the use of CGM in LMIC faces other limitations, including the potential interactions between CGM and certain drugs such as acetaminophen [36], the fact that CGM transmitters can be damaged during computerized tomography/Magnetic Resonance Imaging studies, and the challenges of using such devices in situations of hemodynamic instability.

## 3. Conclusions

The uptake and implementation of CGM devices has significantly altered the management and control of DM wherever it has been implemented, and up-scale of their use for this purpose will likely be beneficial and cost-effective globally, although it is foreseeable that their use in LMICs in the short term will remain anecdotal. In these settings, however, additional public health benefits could be expected if such devices were to be selectively utilized for the monitoring, during hospitalization, of severely ill patients at high risk of developing hypoglycaemia, a common but insufficiently recognized complication of many frequent paediatric conditions which is associated with an excessive and unacceptable risk of death. High quality research evaluating the specific cost-effectiveness of these continuous monitoring devices for altering the prognosis of this life-threatening complication is needed, particularly in relation to their current prohibitive costs, which make them unaffordable for wider general use in settings with many other pressing needs. International consensus recommendations on the use of CGM [3], currently very much focused on their use for DM control, should widen their scope so as to also consider in the future their potential contributing role to the monitoring of hypoglycaemia among severely ill children from LMICs, as this is a common complication that can significantly alter the prognosis of many common conditions in these settings.
